# Metabolic Patterns of Fluconazole Resistant and Susceptible *Candida auris* Clade V and I

**DOI:** 10.3390/jof10080518

**Published:** 2024-07-25

**Authors:** Robab Ebrahimi Barough, Javad Javidnia, Ali Davoodi, Fereshteh Talebpour Amiri, Maryam Moazeni, Shahabeddin Sarvi, Reza Valadan, Ali Siahposht-Khachaki, Mahmood Moosazadeh, Mohsen Nosratabadi, Iman Haghani, Jacques F. Meis, Mahdi Abastabar, Hamid Badali

**Affiliations:** 1 Student Research Committee, Mazandaran University of Medical Sciences, Sari 48157-33971, Iran; robabebrahimibarogh@gmail.com (R.E.B.); nosratabadi.mohsen@yahoo.com (M.N.); 2 Invasive Fungi Research Center, Communicable Diseases Institute, Mazandaran University of Medical Sciences, Sari 48157-33971, Iran; javidniaj@gmail.com (J.J.); moazenimaryam@gmail.com (M.M.); imaan.haghani@gmail.com (I.H.); 3 Department of Medical Mycology, School of Medicine, Mazandaran University of Medical Sciences, Sari 48157-33971, Iran; 4 Department of Pharmacognosy and Biotechnology, School of Medicine, Mazandaran University of Medical Sciences, Sari 48157-33971, Iran; adavoodipharm@gmail.com; 5 Department of Anatomy, Faculty of Medicine, Mazandaran University of Medical Sciences, Sari 48157-33971, Iran; ftaleb2001@yahoo.co.uk; 6 Department of Parasitology, Communicable Diseases Institute, Toxoplasmosis Research Center, Mazandaran University of Medical Sciences, Sari 48157-33971, Iran; shahabsarvi@mazums.ac.ir; 7 Department of Immunology, School of Medicine, Mazandaran University of Medical Sciences, Sari 48157-33971, Iran; valadan.reza@gmail.com; 8 Molecular and Cell-Biology Research Center, Mazandaran University of Medical Sciences, Sari 48157-33971, Iran; 9 Department of Physiology and Pharmacology, Mazandaran University of Medical Sciences, Ramsar International Branch, Sari 48157-33971, Iran; ak57n@yahoo.com; 10 Health Sciences Research Center, Addiction Institute, Mazandaran University of Medical Sciences, Sari 48157-33971, Iran; mmoosazadeh1351@gmail.com; 11 Center of Expertise in Mycology, Radboud University Medical Center, Canisius Wilhelmina Hospital, 6532 SZ Nijmegen, The Netherlands; jacques.meis@gmail.com; 12 Institute of Translational Research, Cologne Excellence Cluster on Cellular Stress Responses in Aging-Associated Diseases (CECAD), Excellence Center for Medical Mycology (ECMM), University of Cologne, 50923 Cologne, Germany; 13 Department of Molecular Microbiology & Immunology, South Texas Center for Emerging Infectious Diseases, The University of Texas at San Antonio, San Antonio, TX 78249, USA

**Keywords:** *Candida auris*, multidrug resistance, gas chromatography, GC-MS, secondary metabolites

## Abstract

*Candida auris*, an emerging non-*albicans* multidrug-resistant yeast, has become a significant cause of invasive candidiasis in healthcare settings. So far, data on the metabolites of *C. auris* in different clades are minimal, and no studies have focused on clade V metabolites. Therefore, Gas chromatography–mass spectrometry (GC-MS) was used for the metabolomic profiling of clade I *C. auris* compared with fluconazole-resistant and susceptible *C. auris* in clade V strains. GC-MS chromatography revealed 28, 22, and 30 compounds in methanolic extracts of the fluconazole-susceptible and fluconazole-resistant *C. auris* clade V and *C. auris* clade I strain, respectively. Some compounds, such as acetamide and metaraminol, were found in fluconazole-susceptible and resistant *C. auris* clade V and clade I. N-methyl-ethanamine and bis(2-ethylhexyl) phthalate metabolites were found in both fluconazole -susceptible and resistant *C. auris* clade V, as well as 3-methyl-4-isopropylphenol, 3,5-bis(1,1-dimethyl)-1,2-benzenediol, and diisostyl phthalate metabolites in both fluconazole resistant *C. auris* clade V and I. Identifying these metabolites contributes to understanding the morphogenesis and pathogenesis of *C. auris*, highlighting their potential role in antifungal drug resistance and the control of fungal growth. However, further experiments are warranted to fully comprehend the identified metabolites’ regulatory responses, and there may be potential challenges in translating these findings into clinical applications.

## 1. Introduction

*Candida* infections pose a serious global threat to human health due to their high mortality rates worldwide [1]. While *Candida albicans* is the most common cause of invasive candidiasis in hospitalized patients, the emergence of non-*albicans Candida* species has increased, likely driven by the widespread prophylactic or therapeutic use of fluconazole [2]. One of the most concerning non-*albicans Candida* species is *Candida auris*, which has demonstrated resistance to fluconazole and other antifungal agents, severely limiting treatment options [3,4,5]. Genomic studies have classified *C. auris* into distinct geographic clades, with four main clades (I–IV) originally identified [4]. A fifth (clade V) was identified in Iran, and a sixth Indomalayan clade has been recently described [6,7]. While clade V is generally fluconazole-susceptible, recently, fluconazole resistance has been reported. Recent reports have even identified cases of fluconazole-resistant *C. auris*, highlighting the urgent need to better understand the underlying mechanisms of resistance in this pathogen. Fungal metabolites are most likely communication signals, allowing them to transmit information and provide some regulatory responses during infection [8,9]. In contrast to *C. albicans*, morphogenetic switching between yeast cells and filaments has not been reported in *C. auris*, and some reports suggest that this species is only capable of producing pseudohyphae [10]. Environmental conditions and the produced metabolites affect the transition between budding yeasts and hyphal growth. Indeed, various metabolites trigger a tightly regulated network of signaling pathways that commonly activate or overexpress some functional genes involving biofilm [11].

The capacity of a fungal cell to withstand environmental stresses and host defenses is profoundly influenced by its metabolic and physiological status and, consequently, by its local nutrient availability [12]. Therefore, the mechanisms of pathogenesis and resistance to the immune system can be understood by a correct and complete understanding of the metabolites produced by an organism. According to various studies, several metabolites, such as farnesol and tyrosol, play critical roles in morphogenetic switching [13]. However, comprehensively profiling the metabolome of a fungal organism is challenging due to the wide range of metabolite concentrations available and the diversity of their biochemical properties [14]. Data on the metabolic profiles of *C. auris* clades and how they may differ from other *C. auris* clades remain extremely limited. Despite this, Brandt et al., using Biolog Phenotype MicroArrays for microbial cells, found clade-specific metabolic differences [15]. Moreover, Viana et al. identified different lipids, carbohydrates, and enzymes using the high-performance liquid chromatography (HPLC) technique. They compared it to the genome-scale metabolic model available for other pathogenic *Candida* species [16]. This research discovered that 50 enzymes were identified as potential drug targets. Elucidating the metabolomic signatures of clade V could provide crucial insights into the remarkable genetic and phenotypic diversity observed across *C. auris* isolates. The current study aimed to analyze the metabolic profiles of fluconazole-susceptible and resistant *C. auris* clade V strains compared with the fluconazole-susceptible *C. auris* clade I isolate.

## 2. Materials and Methods

### 2.1. Isolates and Culture Conditions

Two different clades of *C. auris* were utilized in this study: clade V included two resistant (IFRC2087) [5] and susceptible (IFRC4050) isolates [17] to fluconazole isolated from the ears of Iranian patients with otitis, and clade I included an fluconazole-susceptible isolate as the control. These isolates were cultured separately on Sabouraud dextrose agar (SDA, Merck, Darmstadt, Germany) and were incubated for 48 h at 37 °C prior to the experiment. 

### 2.2. Extraction

*Candida* cells were grown separately for 16 h at 37 °C in a yeast nitrogen base (YNB, Condalab, Madrid, Spain) supplemented with 2% glucose. In total, 5 × 10^6^ cells/mL of *Candida* were collected in sterile falcon tubes [18], after which cell-free supernatants were collected by filtration through 0.2 µm filter paper. We collected the filtered supernatants separately from each *Candida* isolate, extracted their metabolites with methanol, and sonicated them using an ultrasonic water bath at 25 °C for 30 min. Subsequently, the extracts were filtered through 0.22 μm filters, and the solvent was evaporated using a rotary evaporator at 45 °C [13].

### 2.3. Derivatization

Derivatization was used to increase the volatility of metabolites and make them compatible with GC conditions [19]. The filtered extract was treated with 50 µL of N-methyl-N (trimethylsilyl) trifluoroacetamide solution containing 1% trimethylchlorosilane. The mixture was then incubated at 37 °C for two hours to yield the derivatives. Then, the final extract was decanted with hexane three times, and the hexane fraction was obtained for GC analysis.

### 2.4. Analysis by GC-MS

The obtained derivatives of *C. auris* strains were isolated and structurally identified using gas chromatography–mass spectrometry (7890 GC & 5975 Mass, Agilent, Santa Clara, CA, USA). Data acquisition and interception were analyzed using ChemStation software (A.10.02). The samples in 500 µL were injected using an autosampler. The compounds were analyzed at 50 to 250 °C using ten increasing degrees, obtaining the retention time and MS data in the final analysis time. All tests were performed in duplicate. Finally, the data were analyzed, and the structures of the isolated compounds were determined.

## 3. Results

**Figure 1:**
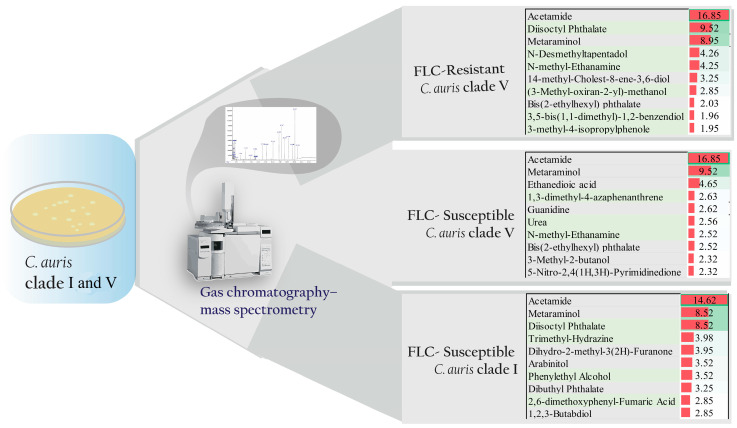
Differential metabolic profiling of fluconazole (FLC)-resistant and FLC-susceptible *Candida auris* clade V strains compared to *C. auris* clade I and V strains. Gas chromatography–mass spectrometry analysis was used to identify and quantify the metabolic profiles produced by the different *C. auris* clades.

**Figure 2:**
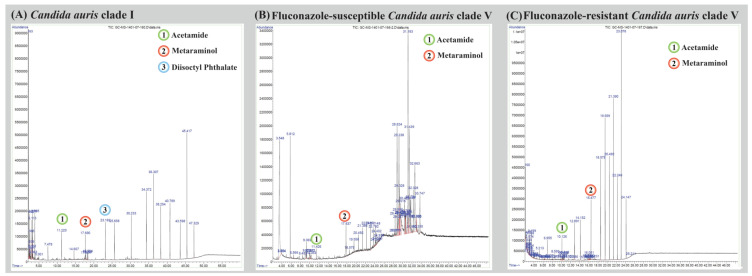
Gas chromatography–MS(GC-MS) chromatograms of *Candida auris* culture extracts were obtained. The chromatograms represent the following strains: (**A**) *C. auris* clade I strain, (**B**) fluconazole-susceptible *C. auris* clade V strain, and (**C**) FLC-resistant *C. auris* clade V strain. The cell-free supernatants were collected and extracted using chloroform. Prior to GC-MS analysis, they were derivatized using an N-methyl-N (trimethylsilyl) trifluoroacetamide solution containing 1% trimethylchlorosilane. The GC-MS analysis was performed using the GC-MS system (7890 GC and 5975 Mass, Agilent, Santa Clara, CA, USA).

Figure 1 illustrates the overall interpretation of the study results. Methanolic extracts of the strains were subjected to GC-MS analysis, and the results showed that the number of compounds detected varied among the different strains. Specifically, 28 compounds were detected in fluconazole-susceptible *C. auris* clade V, 22 in fluconazole-resistant *C. auris* clade V, and 30 in susceptible *C. auris* clade I strains (Figure 2).

The chromatograms in Figure 2 represent the different strains: (A) the susceptible-*C. auris* clade I strain, (B) fluconazole-susceptible *C. auris* clade V strain, and (C) fluconazole-resistant *C. auris* clade V strain. Further analysis of the compounds revealed specific differences between the strains. Fluconazole-susceptible *C. auris* clade V cultures were found to secrete secondary metabolites, such as 4-fluoro histamine-ethanedioic acid, cinnamic acid, urea, guanidine, butanamide, dihydro-4-hydroxy-2(3H)-furanone, 2-methylaminomethyl-1,3-dioxolane, 2,3,5,6-tetramethylphenol, 5-2-methylaminomethyl-1,3-dioxolane, 2-ethylacridine, 1,3-dimethyl-4-azaphenanthrene, and 2-(4-methylphenyl)-indolizine. These metabolites were not found in fluconazole-resistant *C. auris* clade V cultures but were detected in *C. auris* clade I cultures (Figure 2).

Figure 2 GC-MS chromatograms of *Candida auris* culture extracts were obtained. The chromatograms represent the following strains: (A) *C. auris* clade I strain, (B) fluconazole -susceptible *C. auris* clade V strain, and (C) fluconazole-resistant *C. auris* clade V strain. The cell-free supernatants were collected and extracted using chloroform. Prior to GC-MS analysis, they were derivatized using an N-methyl-N (trimethylsilyl) trifluoroacetamide solution containing 1% trimethylchlorosilane. The GC-MS analysis was performed using the GC-MS system (7890 GC and 5975 Mass, Agilent, Santa Clara, CA, USA).

**Figure 3:**
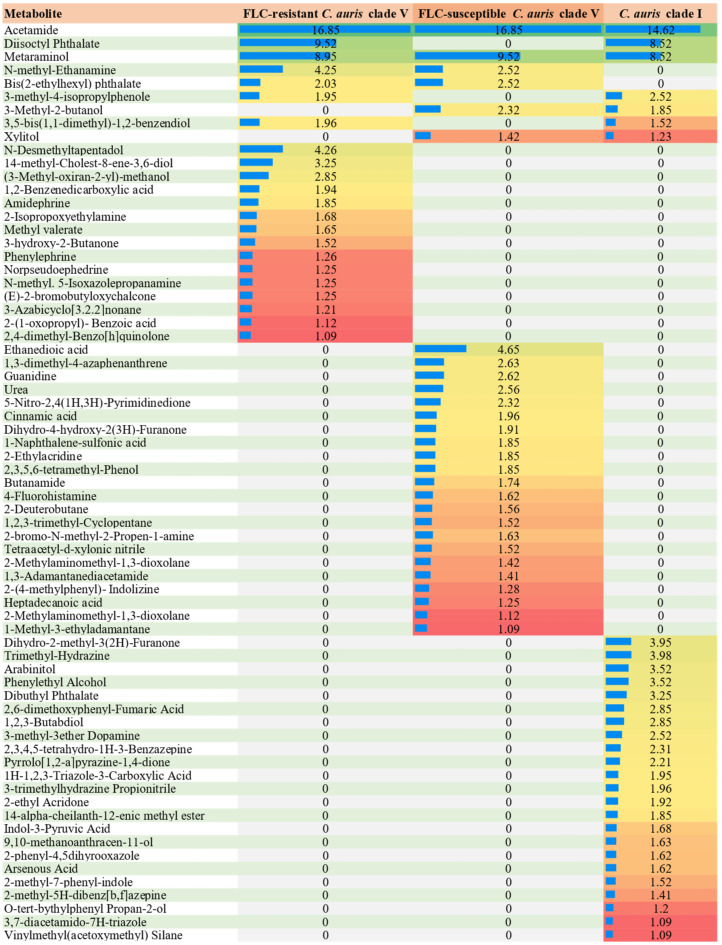
The identified compounds and the percentages of their existence in the extract of fluconazole-resistant and susceptible *Candida auris* clade 5 and *Candida auris* clade 1 culture using gas chromatography–MS are shown.

Figure 3 depicts the identified compounds and the corresponding percentages of their existence in the extracts of fluconazole-resistant and susceptible *C. auris* clade 5 and *Candida auris* clade 1.

On the other hand, compounds such as 1,2,3-butadiol, trimethyl hydrazine, phenylethyl alcohol, triazole-3-carboxylic acid, 1H-1,2,3-triazole-3-carboxylic acid,3-trimethylhydrazine propionitrile, 3,7-diacetamido-7H-triazole, indol-3-pyruvic acid (IPA), 2-phenyl-4,5-dihydrooxazole, vinylmethyl (acetoxymethyl) silane, O-tert-butylphenylpropan-2-ol, 2, 3,4,5-tetrahydro-1h-3-benzazepine, pyrrolo [1,2-a], 2,6-dimethoxyphenyl fumaric acid, 1,4-dibutyl phthalate, pyrazine-1,4-dione, 2-ethyl acridone, ethyl acridone, ethyl arsenic acid, arabinitol, 9,10-methanoanthracen-11-olmethyl ester of 14-alpha-cheilanth-12-enic, and dopamine 3-methyl-3-thiol were found in fluconazole-resistant and fluconazole-susceptible *C. auris* clade V cultures, but not in *C. auris* clade I cultures (Figure 3).

Some compounds, such as acetamide and metaraminol, were found in both fluconazole-susceptible and -resistant *C. auris* clade V and *C. auris* clade I cultures. N-methyl-ethanamine and bis(2-ethylhexyl) phthalate metabolites were identified in both fluconazole-susceptible and resistant *C. auris* clade V cultures, as were 3-methyl-4-isopropylphenol, 3,5-bis(1,1-dimethyl)-1,2-benzenediol and diisostyl phthalate metabolites in both fluconazole-susceptible *C. auris* clade V and *C. auris* clade I.

## 4. Discussion

Metabolomic analysis was performed to identify the cellular metabolome, including intracellular and secreted secondary metabolites. Secondary metabolites secreted by *Candida* species have been implicated in the control of morphogenesis and pathogenesis of these fungi. In particular, several metabolites have been identified as essential factors in inhibiting hyphal formation, promoting biofilm formation, and contributing to antifungal drug resistance. Brandt et al.’s research using Biolog Phenotype MicroArrays identified metabolic differences in *C. auris*. They screened strains from all four previously verified clades on 664 nutrients, 120 chemicals, and 24 stressors. *C. auris* showed robust growth on tricarboxylic acid cycle intermediates but reduced growth on pyruvate, lactic acid, or acetate. The findings emphasize the unique metabolic characteristics of *C. auris*, potentially identifying therapeutic targets [15]. Vienna et al. presented the first validated genome-scale metabolic model (GSMM) for the fungal pathogen *C. auris* named iRV973. The model accurately predicted the growth rate of *C. auris* and its use in different carbon and nitrogen sources. When compared with other yeast models, it was found that 88% of the proteins were shared, indicating common metabolic pathways. There were also 28 exclusive proteins identified in *C. auris*, suggesting unique metabolic features that may have contributed to its emergence as a global health threat [16]. Nonetheless, analytical techniques like mass spectrometry (MS), high-performance liquid chromatography (HPLC), and GC-MS are commonly employed to characterize fungal metabolites. Gas chromatography allows separation based on molecular weight, while MS aids in identifying the metabolites [9,20].

Kadhim et al. [9] and Semreen et al. [21] used GC-MS to identify compounds present in a methanolic extract of *C. albicans*. None of the secondary metabolites secreted by the cultures of *C. albicans*, except for acetamide, were found in *C. auris* clade V or clade I cultures. Metabolomic analysis quantitatively and qualitatively identified the cellular metabolome, including intracellular and secreted secondary metabolites. These metabolites represented the end products of cellular chemical reactions after multiple enzymatic interactions [22].

Several *Candida* species have been found to secrete secondary metabolites, such as hyphal inhibitory metabolites, autoprotective/autotoxic metabolites, and metabolites involved in antifungal drug resistance, including azoles. These metabolites have been identified as essential factors in controlling the morphogenesis and pathogenesis of this fungal species [22,23]. Quorum-sensing molecules such as farnesol, tryptophol, isoamyl alcohol, benzyl alcohol, and phenylethyl inhibit yeast hyphal transformation, while tyrosol promotes filamentation and biofilm formation.

*C. auris*, unlike *C. albicans*, has a round-to-oval yeast morphology and can form pseudohyphae, but not true hyphae, under high salt stress conditions or in biofilms. This difference may be due to the secretion of hyphae-inhibiting metabolites, such as phenylethyl, benzyl, and isoamyl alcohols [24]. Semreen et al. [21] found that *C. auris* strains secreted metabolites, particularly phenylethyl alcohol, benzyl alcohol, isoamyl alcohol, and tyrosol, as well as acids, including benzoic, benzene acetic, glycyrrhizin, and others that were not detected in the *C. albicans* culture. The alcohols phenylethyl, benzyl, and isoamyl were identified as hyphal inhibitory metabolites, whereas tyrosol is a biofilm-forming metabolite.

The presence of phenylethyl, benzyl, and isoamyl alcohols in *C. auris* cultures was consistent. Farnesol was not detected in *C. auris* cultures, but tyrosol, which is required for biofilm formation, was present [21]. The lack of farnesol was observed in both *C. auris* and *C. albicans* cultures when metabolic profiling was performed using GC-MS. Its absence in *C. albicans* cultures was expected as a yeast inhibitory metabolite for hyphae [23]. Farnesol was not found in any *C. auris* clade V or clade I cultures tested. However, since *C. auris* possesses a homolog of *C. albicans* farnesyl synthase, a key enzyme in farnesyl biosynthesis [25], the inability to identify farnesol could be due to the methods’ limitations. This result suggests that the expression of farnesol is not the influential factor for the growth of *C. auris* under the conditions studied, indicating the involvement of other factors in the maintenance of *C. auris* as a yeast.

Interestingly, methyl valerate, a compound used in flavorings and perfumes, was only detected in the metabolites of fluconazole-resistant isolates and has not been reported previously. Amidephrine and metaraminol, both sympathomimetics commonly used to treat allergic rhinitis and hypotension, were found in fluconazole-resistant *C. auris* isolate clade V. This study highlights the importance of environmental conditions in regulating the secretion of aromatic alcohols and emphasizes the need for further investigation into the metabolic mechanisms associated with *C. auris* infections. In addition, the discovery of novel metabolites, such as methyl valerate in fluconazole-resistant isolates, provides insight into the adaptive responses of *C. auris* to antifungal treatment.

Overall, this research improves our understanding of the metabolic landscape of fluconazole-resistant and susceptible *C. auris* clade V strains, paving the way for developing targeted therapeutic interventions against this emerging fungal pathogen. The current study had a limitation regarding the unavailability of all *C. auris* clades, which might have affected the comprehensive understanding of metabolic patterns across different clades. Moreover, just one isolate of *C. auris* clade I was used for comparison. The study specifically focused on clade I and clade V strains, and the absence of other clades restricted the generalizability of the findings to the entire *C. auris* population.

This limitation highlights the importance of future research that includes a broader representation of *C. auris* clades to comprehensively understand the metabolic landscape and its implications for disease control and drug development. Additionally, further research is needed to fully comprehend the identified metabolites’ regulatory responses, and there may be potential challenges in translating these findings into clinical applications. However, a fundamental limitation of the GC-MS methodology used in this study was the inability to detect farnesol, an important quorum-sensing molecule in *Candida* species. Farnesol has been shown to regulate morphological transitions and biofilm formation, which are critical virulence factors for *C. auris.* The volatile and thermolabile nature of farnesol makes it challenging to identify using gas chromatography, which might have resulted in an underrepresentation of this metabolite in the current analysis. Furthermore, a reduced number of *C. auris* strains were examined, which impaired the ability to make robust comparative analyses across clades and resistance profiles. Further research with a larger, more representative sample size is needed to validate and expand upon these preliminary metabolomic findings.

## 5. Conclusions

This study used GC-MS to profile the metabolomes of fluconazole-resistant and susceptible *C. auris* isolates from clade V and clade I strains. The results revealed distinct metabolite signatures between resistance phenotypes and clades, identifying compounds like acetamide, metaraminol, and phthalate derivatives that may play roles in *C. auris* antifungal resistance, morphogenesis, and pathogenesis. Ongoing monitoring of the metabolic diversity within the global *C. auris* population may help elucidate joint adaptability and virulence mechanisms. Translating these insights into practical clinical applications for improved diagnosis, treatment, and infection control remains an important goal for future studies on this emerging multidrug-resistant fungal threat.

## Data Availability

Data are contained within the article.
